# Laser-induced ultrasound transmitters for large-volume ultrasound tomography

**DOI:** 10.1016/j.pacs.2021.100312

**Published:** 2021-11-10

**Authors:** D. Thompson, J.R. Nagel, D.B. Gasteau, S. Manohar

**Affiliations:** Multimodal Medical Imaging (M3i) group, Technical Medical Centre, University of Twente, Netherlands

**Keywords:** Industrial, NDE, Environmental applications of photoacoustics/laser ultrasound, New transducers

## Abstract

We present a protocol for the design, fabrication and characterisation of laser-induced ultrasound transmitters with a specific, user-defined frequency response for the purpose of ultrasound tomography of large-volume biomedical samples. Using an analytic solution to the photoacoustic equation and measurements of the optical and acoustic properties of the materials used in the transmitters, we arrive at a required mixture of carbon black and polydimethylsiloxane to achieve the desired frequency response. After an in-depth explanation of the fabrication and characterisation approaches we show the performance of the fabricated transmitter, which has a centre frequency of 0.9 MHz, 200% bandwidth and 45.8${}^{\circ }$ opening angle, multi-kPa pressures over a large depth range in water.

## 1 Introduction

Laser-induced ultrasound (LIUS) for biomedical imaging applications has been an emerging field of study for the last two decades [1], [2], [3]. LIUS entails the use of ultrasound pulses generated by optical means, using the photoacoustic effect, for ultrasound imaging. In the field of nondestructive materials evaluation it is usual to use the surface of the sample itself as the ultrasound source, as the samples are often similar enough to one another to generate reproducible signals [4], [5]. In biomedical imaging the sample surface is generally much more variable and complex in shape and composition, and safety concerns limit the possible illumination strength, necessitating the use of an external LIUS source. A LIUS transmitter, then, is an engineered optically absorbing object with a specific set of optical and mechanical properties to generate a desired ultrasound field depending on the ultrasound imaging application at hand.

LIUS is preferred for minimally-invasive imaging applications e.g. interstitial imaging [6], [7] owing to the ease with which transmitters can be miniaturised by coating the tip of an optical fibre to create an active element. Another advantage of using LIUS is the ability to avoid inter-element crosstalk when independently exciting many closely spaced ultrasound sources [8], [9], [10], [11], a feature of interest considering the current trend towards miniaturisation of ultrasound multi-element arrays. The potentially very broadband frequency response of LIUS transmitters [12], [13] also promises advances in the fields of acoustic microscopy and mesoscopy.

Ever more sophisticated fabrication methods offer a large degree of control over the LIUS pulse properties [14] by manipulating the backing structure. It has also been shown that dynamic focusing can be achieved by creating a mechanically deformable transmitter [15], as well as combining a LIUS transmitter and broadband detector in a single package [16].

Several applications also make use of the natural combination of LIUS imaging with photoacoustic imaging (PAI) or tomography (PAT) to achieve multimodal acoustic imaging using all-optical excitation. In one such example an optical fibre was fitted with a selectively absorbing film for LIUS excitation and an integrated optical sensor based on a ring resonator, to allow miniaturised PA/LIUS sensing and imaging [17]. In another example a larger-scale tomographic set up was built for combined PAT and LIUS transmission and reflection tomography of small animals [18], giving PA, B-mode and speed-of-sound images of the target.

Most of the applications of LIUS in biomedical imaging from the literature share the characteristic that they tend to focus on imaging relatively small targets like zebrafish and mice, or intravascular imaging. In these cases it is advantageous to use LIUS sources with high optical absorption, to both maximise the generated peak pressure and bandwidth of the generated acoustic pulses to improve spatial resolution. Recent work by Joo et al. [19] looks towards non-invasive applications in larger tissue volumes with the resulting need for a larger penetration depth of the LIUS pulse. Their set up generates focused ultrasound for non-invasive tissue ablation, where the desired treatment depth of multiple centimetres precludes the use of high-frequency, broadband pulses as attenuation scales with frequency in a power law. They achieve this frequency tuning primarily by using thicker absorbing films, up to 1.4 mm in thickness, with lower optical absorption. The combination of the two serves as the main method of controlling the output characteristics of the transmitter.

The current work presents a detailed look at the design, fabrication and characterisation of unfocused LIUS transmitters for large-volume ultrasound CT of, for example, the human breast. The use of LIUS can be beneficial for the prevention of crosstalk, because the transmitters are intended to be used with a high density of highly sensitive PZT detectors. Further, this decoupling of transmission and detection allows for the independent optimisation of both. Since it is important, in this case, to detect the LIUS signal with as many detectors as possible per shot, a large opening angle in addition to a lower centre frequency for transmission through around 10 cm of tissue are desirable features. We present the design considerations in terms of geometry and dimensions and an analytic approach to estimating the output characteristics of such a transmitter. A protocol for fabricating such a transmitter will be presented in detail. We also show the methods used to optically and acoustically characterise the materials in the active element and the response of the finalised transmitters. While the transmitters presented here are designed for a specific imaging application and have the properties to match, the design and fabrication approach can be easily adapted to attain any desired set of transmitter properties using low-cost, readily available materials.

## 2 Materials and methods

### 2.1 Design requirements

The transmitters are designed to match with a reasonable precision to the response and sensitivity of a custom detector (Imasonic SAS, Besançon, France). The detector frequency response peaks at 1 MHz with a bandwidth of roughly 100%. The remaining pressure after transmission across the imaging volume, a distance of some 30 cm, needs to be above the minimum detectable pressure, around 0.5 Pa. An opening angle of at least 30${}^{\circ }$ (1/e) is desired, to match the angular sensitivity of the detectors. A greater opening angle, if sufficient pressure can be maintained, would be beneficial.

For this particular transmitter design the physical dimensions are restricted by the need to use them with an existing set up [20]. The set up encompasses a 30 cm diameter hemispherical imaging volume, surrounded by a pair of 20 mm thick 3D-printed nylon hemisphere segments with universal mounting holes for any desired combination of ultrasound detectors, transmitters and optical fibre bundles for photoacoustic tomography. The mounting holes comprise an initial opening with a 10 mm diameter and an 11.5 mm depth, with the rest of the hole having an 8 mm diameter.

### 2.2 Transmitter design

**Fig. 1:**
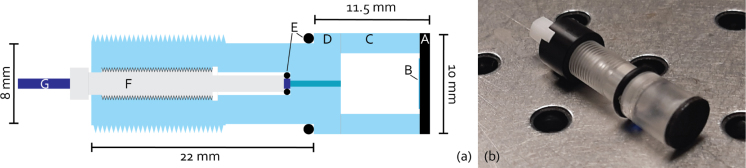
(a) Schematic representation of the transmitter, including essential outer dimensions and individual components. (A) Optically absorbing active element, (B) High-impedance backing layer, (C) Tube segment enclosing air cavity, (D) Threaded mounting piece with fibre passage, (E) Outer and inner O-rings for watertightness and fibre clamping (inner only), (F) Fibre clamping chuck, (G) Optical fibre for light delivery. (b) Photograph of a finished LIUS transmitter.

Fig. 1(a) shows the design and resulting dimensions, the different components are also labelled. The dimensions of elements D and F in Fig. 1 are fixed, while the thickness of the active element, component A, can be varied depending on the required optical and acoustic properties, with the length of component C being allowed to vary accordingly so as to maintain the overall required dimensions. In response to the design constraints the transmitter is cylindrical in shape, with a 10 mm diameter head of 11.5 mm length and a tail of 8 mm diameter and 22 mm length. The final 15 mm of the transmitter are threaded, to allow easy fastening in the mounting hole with a nut.

### 2.3 Analytical model for LIUS generation

The properties of the generated acoustic pulse can be influenced, within our design, by the optical absorption coefficient $\mu_{a}$ [${\text{m}}^{-1}$], the dimensions and fluence of the projected light spot and the dimensions of the active element. To be able to guide the fabrication process, it is important to be able to predict the behaviour of a transmitter with some accuracy when the above-mentioned properties are varied. To this end, an analytic expression for the generated signal as a function of the design parameters is derived in the following.

The pressure detected at a given point as a function of time can be described as follows, by solving the photoacoustic equation with the Green’s function approach for an optical delta pulse [21], [22]: (1)
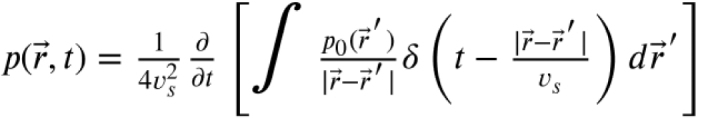
In cylindrical coordinates with the origin at the centre of the illumination spot on the back surface of the absorber:

(2) $$p_{0}\left(r^{\prime },z,\theta \right)=\frac{\Gamma \mu_{a}\phi_{0}\eta_{th}}{4v_{s}^{2}}\left(e^{\frac{-r^{\prime 2}}{2w_{0}^{2}}-\mu_{a}z}\Pi \left(z-\frac{\kappa }{2}\right)+R_{pg}e^{\frac{-r^{\prime 2}}{2w_{0}^{2}}+\mu_{a}z}\Pi \left(z+\frac{\kappa }{2}\right)\right)\left(+\underset{n}{\sum }T_{pg}R_{ga}^{n}R_{gp}^{n-1}T_{gp}e^{\frac{-r^{\prime 2}}{2w_{0}^{2}}+\mu_{a}\left(z+n\delta \right)}\Pi \left(z+\frac{\kappa +n\delta }{2}\right)\right)$$

The first term is due to the direct pulse from the initial pressure distribution while the second term denotes its reflection off the back surface of the absorber, with $R_{pg}$ the coefficient of reflection between the active element and the backing material. The third term is a summation of all the internal reflections in the backing material, with $T_{pg}$ denoting the pressure transmission coefficient into the backing, $R_{ga}$ the reflection coefficient off the interface with the air pocket, $R_{gp}$ the reflection coefficient at the interface between backing and active element and $T_{gp}$ the transmission coefficient at the same location. Each consecutive internal reflection gives rise to a virtual source shifted by a distance nδ, where δ depends on the sound speed and thickness of the backing material and the sound speed in the active element. Additionally, $\eta_{th}$ denotes the thermal conversion efficiency, r, $w_{0}$ and z are the radial coordinate, $e^{-1}$ width of the laser spot and depth into the absorber respectively, all in m, and finally $\phi_{0}$ [$\text{J}\;{\text{m}}^{-2}$] is the light fluence at the origin. The gate functions that ensure this behaviour are defined as follows:

(3) $$\Pi \left(z\pm \frac{\kappa }{2}\right)=\left\{\begin{array}{c} 0\;\text{if}\;\left|z\pm \frac{\kappa }{2}\right|>\frac{\kappa }{2}\;\\ 1\;\text{if}\;\left|z\pm \frac{\kappa }{2}\right|\leq \frac{\kappa }{2}\; \end{array}\right)$$

Here, κ denotes the thickness of the absorbing layer. The dimensionless Grüneisen parameter is defined as follows:

(4) $$\Gamma =\frac{\beta }{\kappa \rho C_{V}}$$

where β [${\text{K}}^{-1}$] is the thermal expansion coefficient, κ [${\text{Pa}}^{-1}$] the isothermal compressibility and ρ [$\text{kg}\;{\text{m}}^{-3}$] and $C_{V}$ [$\text{J}\;{\text{kg}}^{-1}\;{\text{K}}^{-1}$] the mass density and isochoric specific heat capacity, respectively.

**Fig. 2:**
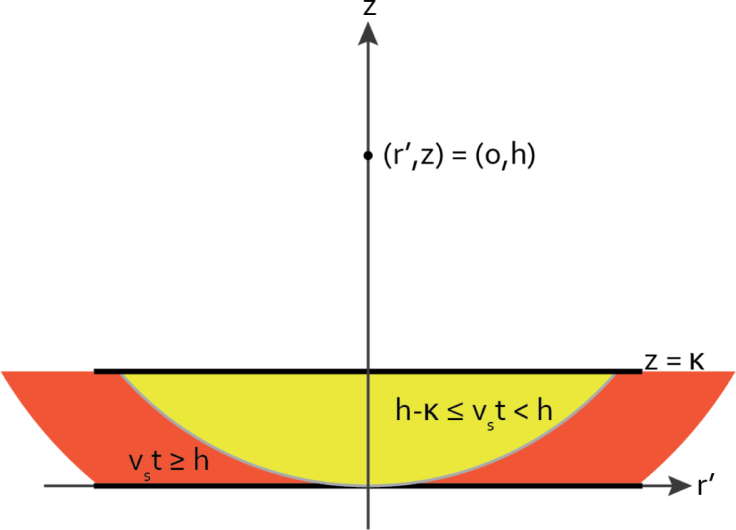
Schematic of the geometry of the analytical problem, showing the lower surface of the absorber at z=0, the upper surface at z=κ, detector on the acoustic axis at z=h, and the different integration regions for the direct pulse.

In the following we assume the laser spot size to be significantly smaller than the diameter of the absorber, which is thus treated as though it extends infinitely in the lateral dimension $r^{\prime }$. For a detector placed on the acoustic axis at a distance z=h from the origin as shown in Fig. 2, Eq. (1) then becomes: (5)
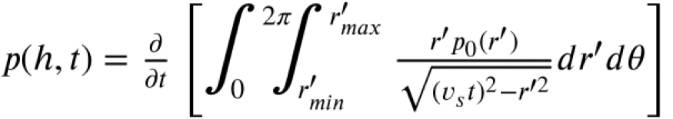
The integral over θ simply yields a factor 2π. By changing variables in the integral to $\alpha =\sqrt{{\left(v_{s}t\right)}^{2}-r^{\prime 2}}$ and adding the explicit expression for $p_{0}$ this results in the following series of Gaussian integrals: (6)
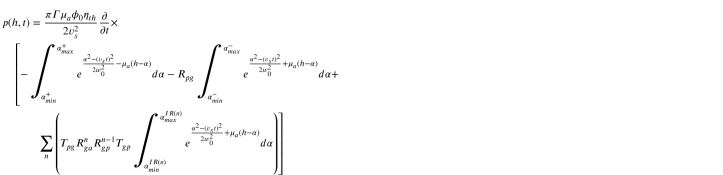
The first integral describes the pulse propagating directly towards the detector, the second is the contribution from the reflection off the backing and the third term is the total contribution of any internal reflections in the backing. The integration limits as function of time and detector position are

(7) $$\left[\alpha_{min}^{+},\alpha_{max}^{+}\right]=\left\{\begin{array}{cc} \;\left[v_{s}t,h-\kappa \right]\; & \text{for}\;h-\kappa \leq v_{s}t\leq h\\ \;\left[h,h-\kappa \right]\; & \text{for}\;v_{s}t>h \end{array}\right)$$

$$\left[\alpha_{min}^{-},\alpha_{max}^{-}\right]=\left\{\begin{array}{cc} \;\left[v_{s}t,h\right]\; & \;\text{for}\;h\leq v_{s}t\leq h+\kappa \\ \;\left[h+\kappa ,h\right]\; & \;\text{for}\;v_{s}t>h+\kappa \end{array}\right)$$

$$\left[\alpha_{min}^{I\left(n\right)},\alpha_{max}^{I\left(n\right)}\right]=\left\{\begin{array}{cc} \;\left[v_{s}t,h+n\delta \right]\; & \;\text{for}\;h+n\delta \leq v_{s}t\leq h+\kappa +n\delta \\ \;\left[h+\kappa +n\delta ,h+n\delta \right]\; & \;\text{for}\;v_{s}t>h+\kappa +n\delta \end{array}\right)$$

The two ranges of $v_{s}t$ values for the direct pulse are illustrated in Fig. 2. Performing the integration and differentiation in Eq. (6) leads to three sets of equations in terms of Dawson functions [23], for the direct forward propagating pressure $p_{d}$:

(8) $$p_{d}\left(h,t\right)=A\left\{\begin{array}{cc} \begin{array}{cc} & (v_{s}e^{-\mu_{a}\left(h-v_{s}t\right)}\left(\left(\mu_{a}-2X_{1}\right)D\left(X_{1}\right)+\frac{1}{\sqrt{2}w_{0}}\right)\\ & \;+\frac{v_{s}^{2}t}{w_{0}^{2}}e^{\frac{{\left(h-\kappa \right)}^{2}-{\left(v_{s}t\right)}^{2}}{2w_{0}^{2}}-\mu_{a}\kappa }D\left(X_{2}\right)) \end{array}\; & for\;h-\kappa \leq v_{s}t<h\\ \begin{array}{cc} \frac{v_{s}^{2}t}{w_{0}^{2}} & \left(e^{\frac{{\left(h-\kappa \right)}^{2}-{\left(v_{s}t\right)}^{2}}{2w_{0}^{2}}-\mu_{a}\kappa }D\left(X_{2}\right)-e^{\frac{h^{2}-{\left(v_{s}t\right)}^{2}}{2w_{0}^{2}}}D\left(X_{3}\right)\right) \end{array}\; & for\;v_{s}t\geq h \end{array}\right)$$

for the reflection of the interface between active element and backing, $p_{pg}$:

(9) $$p_{pg}\left(h,t\right)=AR_{pg}\left\{\begin{array}{cc} \;0\; & for\;v_{s}t<h\\ \begin{array}{cc} & (v_{s}e^{\mu_{a}\left(h-v_{s}t\right)}\left(\left(\mu_{a}-2\bar{X_{1}}\right)D\left(\bar{X_{1}}\right)+\frac{1}{\sqrt{2}w_{0}}\right)\\ & \;+\frac{v_{s}^{2}t}{w_{0}^{2}}e^{\frac{h^{2}-{\left(v_{s}t\right)}^{2}}{2w_{0}^{2}}}D\left(\bar{X_{3}}\right)) \end{array}\; & for\;h\leq v_{s}t<h+\kappa \\ \begin{array}{cc} \frac{v_{s}^{2}t}{w_{0}^{2}} & \left(e^{\frac{{\left(h+\kappa \right)}^{2}-{\left(v_{s}t\right)}^{2}}{2w_{0}^{2}}-\mu_{a}\kappa }D\left(\bar{X_{2}}\right)-e^{\frac{h^{2}-{\left(v_{s}t\right)}^{2}}{2w_{0}^{2}}}D\left(\bar{X_{3}}\right)\right) \end{array}\; & for\;v_{s}t\geq h+\kappa \end{array}\right)$$

and finally for the contributions from internal reflections in the backing layer, $p_{ga}$:

(10) $$p_{ga}\left(h,t\right)=A\left\{\begin{array}{c} \;0\;\\ \;for\;v_{s}t<h+\delta \;\\ \begin{array}{cc} & \overset{\left\lfloor \frac{v_{s}t-h}{\delta }\right\rfloor }{\underset{n=1}{\sum }}T(v_{s}e^{\mu_{a}\left(h-v_{s}t\right)}\left(\left(\mu_{a}-2\bar{\bar{X_{1}}}\right)D\left(\bar{\bar{X_{1}}}\right)+\frac{1}{\sqrt{2}w_{0}}\right)\\ & \;+\frac{v_{s}^{2}t}{w_{0}^{2}}e^{\frac{{\left(h+n\delta \right)}^{2}-{\left(v_{s}t\right)}^{2}}{2w_{0}^{2}}}D\left(\bar{\bar{X_{3}}}\right)) \end{array}\;\\ \;for\;h+\delta \leq v_{s}t<h+\kappa +\delta \;\\ \begin{array}{cc} & \overset{\left\lfloor \frac{v_{s}t-h-\kappa }{\delta }\right\rfloor }{\underset{n=1}{\sum }}T\frac{v_{s}^{2}t}{w_{0}^{2}}\left(e^{\frac{{\left(h+\kappa +n\delta \right)}^{2}-{\left(v_{s}t\right)}^{2}}{2w_{0}^{2}}-\mu_{a}\left(\kappa +n\delta \right)}D\left(\bar{\bar{X_{2}}}\right)-e^{\frac{h^{2}-{\left(v_{s}t\right)}^{2}}{2w_{0}^{2}}}D\left(\bar{\bar{X_{3}}}\right)\right)\\ & \;+\;\overset{\left\lfloor \frac{v_{s}t-h}{\delta }\right\rfloor }{\underset{n=\left\lfloor \frac{v_{s}t-h-\kappa }{\delta }\right\rfloor }{\sum }}T(v_{s}e^{\mu_{a}\left(h-v_{s}t\right)}\left(\left(\mu_{a}-2\bar{\bar{X_{1}}}\right)D\left(\bar{\bar{X_{1}}}\right)+\frac{1}{\sqrt{2}w_{0}}\right)\\ & \;+\frac{v_{s}^{2}t}{w_{0}^{2}}e^{\frac{{\left(h+n\delta \right)}^{2}-{\left(v_{s}t\right)}^{2}}{2w_{0}^{2}}}D\left(\bar{\bar{X_{3}}}\right)) \end{array}\;\\ \;for\;v_{s}t\geq h+\kappa +\delta \; \end{array}\right)$$

Where the following constants and variables are defined, for compactness’ sake:

(11) $$A=\frac{w_{0}\pi \Gamma \mu_{a}\phi_{0}\eta_{th}}{\sqrt{2}v_{s}^{2}};T=T_{pg}R_{ga}^{n}R_{gp}^{n-1}T_{gp};$$

$$X_{1}=\frac{\mu_{a}w_{0}^{2}+2v_{s}t}{2w_{0}};X_{2}=\frac{\mu_{a}w_{0}^{2}+2\left(h-\kappa \right)}{2w_{0}};\;X_{3}=\frac{\mu_{a}w_{0}^{2}+2h}{2w_{0}}$$

$$\bar{X_{1}}=\frac{\mu_{a}w_{0}^{2}-2v_{s}t}{2w_{0}};\bar{X_{2}}=\frac{\mu_{a}w_{0}^{2}-2\left(h+\kappa \right)}{2w_{0}};\;\bar{X_{3}}=\frac{\mu_{a}w_{0}^{2}-2h}{2w_{0}}$$

$$\bar{\bar{X_{1}}}=\bar{X_{1}};\;\bar{\bar{X_{2}}}=\frac{\mu_{a}w_{0}^{2}-2\left(h+\kappa +n\delta \right)}{2w_{0}};\bar{\bar{X_{3}}}=\bar{X_{3}}$$

Evaluating Eqs. (8)–(10) for a set of $\mu_{a}$ values and absorber thicknesses will produce analytical time signals, of which the frequency spectra can also be calculated numerically. Examining the resulting spectra and pressure values will inform the fabrication process and assist in choosing the proper values for $\mu_{a}$, the absorber thickness and the laser pulse energy.

### 2.4 Fabrication protocol

#### 2.4.1 Fabrication materials

The base material for the LIUS transmitters is polydimethylsiloxane (PDMS, Sylgard 184, Dow Corning, USA), which is mixed with an appropriate amount of carbon black (CB, Printex 60, Palmer Holland, USA) for optical absorption, 45 g of PDMS with 3.63 g of CB for a weight percentage of 8.07%. The transmitter casings are made of machined polymethylmethacrylate (PMMA), and the backing consists of a disc-shaped borosilicate glass microscopy cover slip with a 5 mm diameter and 100μm thickness (Agar Scientific, UK). The various parts are bonded together using a cyanoacrylate-based adhesive (Loctite, Germany).

#### 2.4.2 Fabrication protocol

**Fig. 3:**
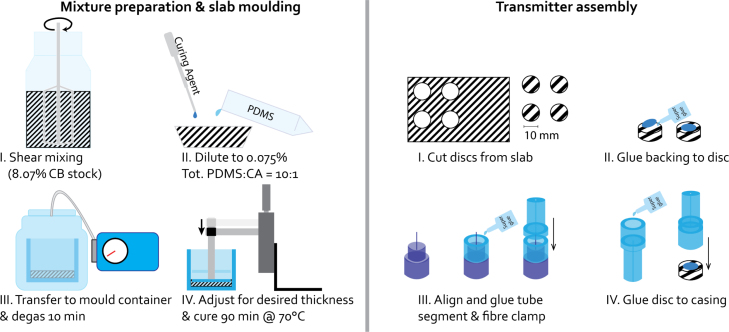
Making a slab of CB/PDMS of the right concentration from a high-concentration stock and assembling it into a LIUS transmitter.

Fig. 3 shows an illustration of the fabrication steps, from the preparation of the absorber material to the assembly of the transmitters themselves. To expedite fabrication of a range of samples in the appropriate range of CB-percentages, and thus optical absorption coefficients, a stock solution with a high concentration of CB was first prepared. This serves the twofold purpose of minimising the requirement for handling CB powder and increasing the accuracy of the amounts weighed, by simply taking larger amounts. By placing an amount of CB powder in a pre-weighed, sealed container and weighing it one can minimise contamination of equipment and loss of CB. By pouring the viscous liquid PDMS on top of the powder and again weighing, the weight percentage of CB in the stock solution is determined. A homogeneous mixture is achieved by mechanical mixing (RW16 basic, IKA Labortechnik, Germany) at 500 rpm for 90 min, breaking up any CB aggregates by the applied shear forces from the flow of the viscous mixture [24].

Next, in a separate container or dish, a desired amount of stock mixture is weighed, denoted $m_{stock}$. Of this weight $m_{0}^{CB}=C_{stock}m_{stock}$ is the weight of carbon black contained in the stock. Eq. (12) can then be used to determine the weight of clear PDMS, denoted $m_{PDMS}$, to be added to achieve the desired weight fraction of CB, $C_{final}$, based on the weight fraction of CB in the stock mixture ($C_{stock}$), assuming a 10:1 weight ratio of PDMS to curing agent.

(12) $$m_{PDMS}=\frac{\frac{m_{0}^{CB}}{C_{final}}-1.1\left(1-C_{stock}\right)m_{stock}}{1.1}$$

After adding the required weight of clear PDMS a disposable plastic pipette is used to add curing agent of the desired 10% of the total PDMS weight, after which it must be well mixed with a small spatula until it appears homogeneous, as the volume is too small to use a mechanical mixer. To facilitate the production of multiple transmitters the mixture is cured in a slab by transferring it to a flat-bottomed rectangular container, degassing in a vacuum vessel at −1 bar and regulating the thickness with a PMMA plate on a translation stage. Any excess liquid will flow past the edge of the plate and is cut off after curing. The mould is then placed in an oven at 70 °C for 90 min to cure.

Once cured and cooled, 10 mm diameter discs are cut from the slab with a mechanical hole punch and a hammer. A small drop of cyanoacrylate adhesive is deposited on one face of a cut disc and smeared out into a thin layer with a needle tip. A glass backing disc is gently but firmly pressed onto the glue for tight adhesion to the disc. The tube segment and fibre mount need to be bonded coaxially, for which purpose a cylindrical alignment piece was machined, shown in image III on the right-hand side of Fig. 3. The centre of the alignment piece contains the cut-off end of one of the optical fibres, which fits snugly into the hollow of the fibre mount, ensuring alignment of the pieces. Cyanoacrylate adhesive is once again deposited and smeared out over the exposed rim of the tube segment, after which the mounting piece is firmly pressed against it and allowed a minute to set. Finally, adhesive is applied in the same manner as the previous steps to the open side of the tube segment, which is then pressed gently but firmly against the CB/PDMS disc and left for a few minutes to dry. After addition of the external and internal o-rings and insertion of an optical fibre the transmitter is now ready for use.

### 2.5 Characterisation methods

Before fabrication of the transmitters could begin, the dependence of the optical properties of the CB/PDMS mixture in the concentration of CB had to be determined. Once known, the acoustic properties of a sample with the required optical properties also had to be characterised. This section will describe the various characterisation approaches taken.

#### 2.5.1 Materials characterisation

The required weight concentration of CB is determined by measuring the optical transmission spectrum for a range of samples of PDMS with CB contents between 0.03% and 0.28%, beyond which the transmitted light is insufficient for an accurate measurement. Any higher concentrations could not be measured by the spectrophotometer (UV2600, Shimadzu, Japan). The samples are prepared by mixing a range of concentrations from the previously prepared stock solution and placing a drop inside a circular, 120μm spacer (SecureSeal, Grace Bio-Labs, USA) in between two microscope slides. By measuring the transmittance over a wavelength range from 400 to 1100 nm, $\mu_{a}$ can be determined for any illumination wavelength within that range and a wide range of CB concentrations, by taking a linear fit to the data. The scattering coefficient of the mixture can be estimated from the mean CB particle size of about 21 nm [25] and the volume fraction of CB in the mixture for any given concentration. We assume Rayleigh scattering [26], as the particle size is significantly smaller than ≈10% of the wavelength of 1064 nm. Using the refractive index and density for CB of 2.00 and 1.8 $\text{kg}\;{\text{m}}^{-3}$ respectively [27] and a value of 1.41 for the refractive index of PDMS, the mean scattering cross section of a CB particle is :

(13) $$\sigma =\frac{8\pi }{3}{\left(\frac{2\pi n_{PDMS}}{\lambda }\right)}^{4}d^{6}{\left(\frac{m^{2}-1}{m^{2}+2}\right)}^{2}$$

where $m=\frac{n_{CB}}{n_{PDMS}}$, *d* is the particle diameter and λ the wavelength of the light. The scattering cross section is multiplied by the number density of CB to obtain the scattering coefficient $\mu_{s}$, which is on the order of 10^−20^ mm^−1^ for the CB concentrations in the range of interest, many orders of magnitude below $\mu_{a}$, and thus negligible.

Once the proper CB-percentage for the desired photoacoustic response is known, a precursor slab for a desired number of transmitters is fabricated according to the description in Section 2.4.2. By making an excess of material for the slab, a block for measurement of the acoustic properties can also be produced. This excess material is poured to a depth of roughly 25 mm in a 50 × 35 × 30 mm${}^{3}$ polypropylene container and cured with the slab to produce a block measuring 50.0 × 34.9 × 25.0 mm${}^{3}$ as measured with a Vernier caliper. Through-transmission ultrasound measurements of the block are performed at frequencies of 1, 2.25, 5, 7.5 and 15 MHz produced by single element transducers (Olympus Panametrics NDT, USA) and measured by a 1 mm diameter PVDF needle hydrophone (Precision Acoustics, UK). From the transmission ultrasound measurements the material sound speed can be determined from the differences in arrival times when measuring through different thicknesses of material. Comparing the signal arrival times between different sides of the block, as well as comparing it to a reference measurement in water then lets us determine the speed of sound [28]:

(14) $$v_{s}=\frac{\delta }{\Delta t\frac{\delta }{v_{ref}}}$$

In Eq. (14), δ [m] denotes the difference in sample thickness between measurements, Δt is the difference in arrival time between two signals and $v_{ref}$ [m s^−1^] is the speed of sound in water.

While the acoustic attenuation between roughly 0 and 4 MHz can be determined from transmission measurements on this block, it was deemed preferable to measure it over a broader frequency range. For this purpose a pair of disc-shaped samples of 2.5 cm diameter and 3.97 and 5.97 mm thicknesses was also cast. In this manner the sound speed can be measured with greater accuracy due to the smaller relative uncertainty in the measured sample thickness, while the attenuation will be known over a broader frequency range. The acoustic attenuation in dB per cm as a function of frequency can be determined from [28]:

(15) $$\alpha \left(f\right)=\frac{20}{\delta }{\text{log}}_{10}\left(\frac{A_{2}\left(f\right)}{A_{1}\left(f\right)}\right)$$

where $A_{2}\left(f\right)$ is the amplitude spectrum of the signal through the thinner sample, $A_{1}\left(f\right)$ the same for the thicker sample. The frequency dependence of the acoustic attenuation generally follows a power law, so a fit to the data can be found in the form:

(16) $$\alpha \left(f\right)=\alpha_{0}f^{b}$$

Once the values of $\alpha_{0}$ and *b* are known they can then be used to estimate the impact of acoustic attenuation on a transmitter of a given thickness.

#### 2.5.2 Transmitter response measurements

A fibre-optic needle hydrophone system (Precision Acoustics, UK) is used to characterise the response of the transmitter under illumination by a 3.6 mm diameter (1/e) Gaussian spot from a compact diode-pumped pulsed Nd:YAG laser (M-NANO PR147, Montfort Laser, Austria) emitting 4.7 ns pulses at a 100 Hz repetition rate at a 1064 nm wavelength. The light from the laser is coupled into a multimode optical fibre (FT600UMT, Thorlabs, USA), in a fibre multiplexer described in more detail in previous work [8]. The hydrophone is placed on a 3D translation stage to enable characterisation of the full acoustic field that is emitted. The temporal and frequency response as well as the field geometry are characterised, emphasising opening angle and on-axis pressure decay. By increasing the laser pulse energy the response of the transmitter to optical fluence, as well as the optical damage threshold, can be found. The on-axis response is also measured using the custom-built single element transducer mentioned in Section 2.2 for comparison.

## 3 Results and discussion

### 3.1 Analytical results

**Fig. 4:**
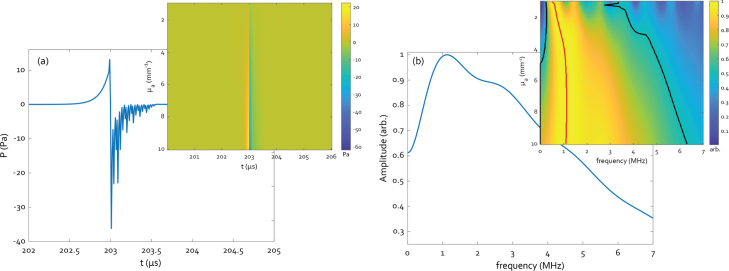
(a) Calculated temporal response for $\mu_{a}=6.4$ mm^−1^ at a depth of 300 mm, exhibiting 49.3 Pa peak-to-peak pressure, inset shows the response of a 1 mm thick absorber as function of $\mu_{a}$. (b) Spectrum for $\mu_{a}=6.4$ mm^−1^, with an inset showing spectra as function of $\mu_{a}$. The red line follows the maximum amplitude, the black lines encompass the −6 dB cut-offs of the spectra.

Evaluating Eqs. (8)–(10) for a glass-backed PDMS (Γ≈0.71) active element with values of $\mu_{a}$ between 1 mm^−1^ and 10 mm^−1^ at a depth of 300 mm with an optical pulse energy of 1 mJ and $w_{0}=2.0$ mm, or a fluence of 7.95 mJ cm^−2^ gives the signals and spectra shown the insets in Fig. 4, for 1 mm active element thickness. The maximum of the spectrum occurs at 1 MHz for a value of $\mu_{a}=6.4$ mm^−1^, with a bandwidth from 0 to 5.5 MHz and peak-to-peak pressure of 49.3 Pa at 300 mm in water. The signal and frequency spectrum for $\mu_{a}=6.4$ mm^−1^ are shown in Fig. 4(a) and (b) respectively. The negative part of the time signal contains a series of diminishing oscillations, the source of which are the internal reflections in the backing layer described by Eq. (10). As acoustic attenuation was not taken into account for these calculations, a somewhat smaller bandwidth is to be expected in the experimental results. With the values extracted from these calculations, the next step is to determine the weight fraction of CB required to achieve a $\mu_{a}$ of 6.4 mm^−1^.

### 3.2 Materials characterisation

#### 3.2.1 Optical properties

**Fig. 5:**
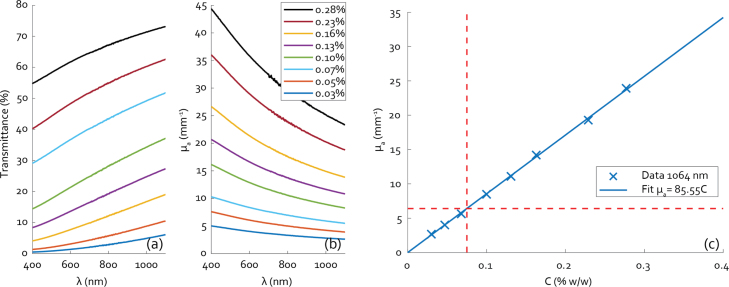
(a) Plots of measured transmittance for a range of CB concentrations in PDMS, (b) the absorption coefficient derived therefrom and (c) $\mu_{a}$ vs concentration at 1064 nm wavelength, including a linear fit to the data. The dashed red lines indicate the desired $\mu_{a}$ value and the corresponding concentration of 0.075% CB by weight.

Fig. 5 shows plots of the transmittance and the corresponding $\mu_{a}$ over a wavelength range from 400–1100 nm. The sample CB concentrations range from 0.03% to 0.28%. Fig. 5(c) shows the measured values of $\mu_{a}$ for the full range of concentrations, denoted C, and a linear fit to the data at λ= 1064 nm. The fit indicates that the desired $\mu_{a}$-value of 6.4 mm^−1^ requires a CB concentration of 0.075% by weight. Calculating the expected value of the scattering coefficient $\mu_{s}$ using the approach delineated in Section 2.5 yields a value of $1.3\cdot 10^{-20}$ mm^−1^, 20 orders of magnitude below $\mu_{a}$ and thus of little consequence to the behaviour of the transmitter.

#### 3.2.2 Acoustic properties

**Fig. 6:**
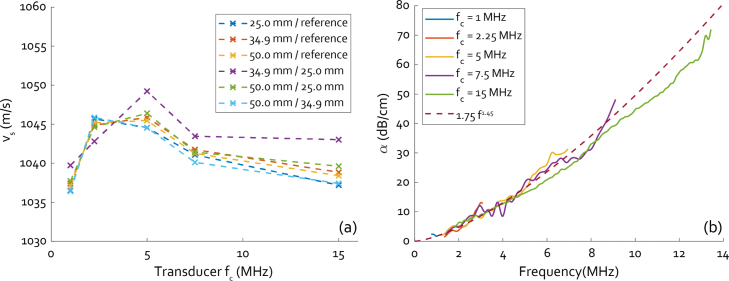
(a) Through-transmission speed-of-sound measurements based on 5 single element transducers, the different coloured curves specify through which side of the block the signal was acquired and to what measurement it was referenced. (b) Attenuation measurements based on comparison of the received spectra through samples of 2 different thicknesses, 3.97 mm and 5.97 mm, using the same set of 5 transducers. The dashed line shows a mean of the fits to the individual measurements.

Fig. 6(a) shows speed-of-sound results of the through-transmission ultrasound measurements of the large block. The different data sets show sound speed values derived from a comparison of a measurement through one side of the block to a reference measurement through water or a measurement through one of the other sides. The sound speed values all cluster together, except for the measurements through the 34.9 mm side referenced to the 25.0 mm side (the purple curve). This discrepancy may be due to the lower reliability of the measured thickness of the 25.0 mm side. The 25.0 mm side includes the top surface of the PDMS which was exposed to the air while curing and exhibits a concave meniscus which may have led to a slight overestimation of the thickness. However, no such noticeable deviation can be seen for the green curve, which is the measurement through the 50.0 mm side referenced to the short side. Overall, from these measurements the sound speed in the 0.075% CB/PDMS material is 1041.78 ± 3.60 m/s, in line with literature values for clear PDMS [28].

Fig. 6(b) shows the attenuation results of the thinner samples. The attenuation curves measured by the different transducers all appear to fall on a similar trend, providing reliable data between 0.64 MHz and 12.84 MHz. Taking the mean values of the fitted parameters of the power law in Eq. (16) gives us $\alpha_{0}=1.75$ ${\text{dB cm}}^{-1}{\text{ MHz}}^{-1}$ and b=1.45, in line with values found in literature [29], [30].

### 3.3 Transmitter performance

**Fig. 7:**
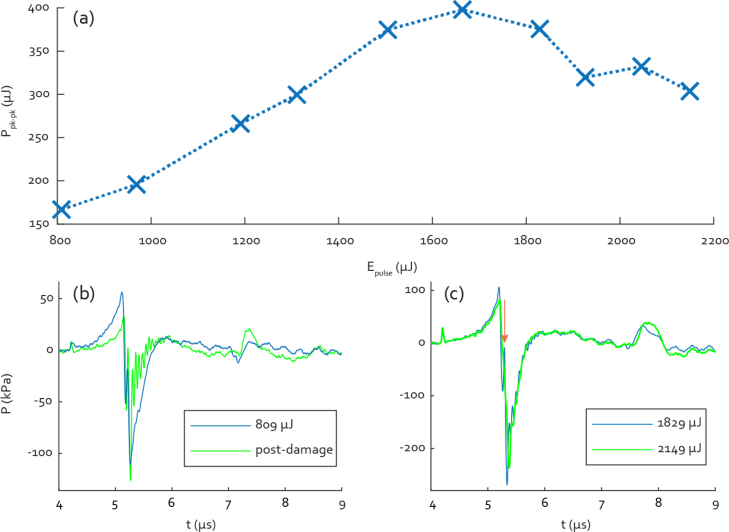
(a) Overview of peak-to-peak LIUS pulse pressures with increasing illumination pulse energy, showing a clear levelling off and subsequent decrease due to damage to the back face of the transmitter.(b) Comparison of LIUS pulse at 809 μJ illumination before and after laser-induced damage. (c) LIUS pulses when illuminating at 1829 and 2149 μJ, showing appearance and subsequent disappearance of oscillations in the rising edge of the negative peak.

The generated peak-to-peak pressure was measured using illumination pulse energies between 809 μJ and 2149 μJ, or fluence values between 7.9 and 21.1 mJ cm^−2^, at a distance of 6.2 mm from the transmitter surface. An overview is shown in Fig. 7(a), the pressure increases linearly from 166.8 kPa to 398.1 kPa between 809 and 1664 μJ, after which it seems to decrease in a more or less linear fashion. The time signals started deforming at illumination pulse energies in excess of 1505 μJ ($\phi_{0}=14.8$ mJ cm^−2^), exhibiting oscillations in the rising flank of the negative peak currently suspected to indicate detachment of the backing from the absorbing layer. There appear to be no oscillations visible in the pre-damage curve in Fig. 7(b), in contrast to the simulation results in Fig. 4(a). This could be due to the intervening layer of cyanoacrylate adhesive acting as a matching layer, smoothing out the signal. Optical damage to the glue layer may then have changed the acoustic properties and thickness of the bond, introducing the oscillations from internal reflections in the backing, which can be seen clearly in the green curve in Fig. 7(b). Both signals in Fig. 7(b) were measured at an illumination pulse energy of 809 μJ, the post-damage curve being measured after going through the full range of pulse energies up to 2150 μJ. All signals for this particular transmitter, up to a pulse energy of 1664 μJ, also exhibited a positive spike in the middle of the falling edge of the positive peak (red arrow in Fig. 7(c)), most likely due to an excess of glue between the glass and the CB/PDMS. As can be seen in Fig. 7(c) this spike disappears at pulse energies of 1829 μJ and over, as do the rising-flank oscillations, though both reappear at lower energies. The higher-energy signal still has a bulge at the spike location and the negative peak has shifted to the right by 36 ns. The positive temporal shift of the negative peak would indicate a further increase in the distance between the PDMS and the glass backing, which would shift any signals due to reflection off the backing to the right. As the effect seems reversible it is likely to be a thermal expansion effect in the cyanoacrylate layer.

The detachment may well have worsened at higher pulse energies, partially explaining the decrease in generated pressure. At higher pulse energies the amplitude of the internal-reflection peak at t=8μs also increased, this persisted when illuminating at lower energies again, as seen in Fig. 7(a). Upon disassembly of the damaged transmitter the backing appeared white and optically opaque, likely due to damage in the cyanoacrylate layer. This opacity may well be the main cause for the decreased amplitude, while damage-induced changes in the acoustic properties of the glue could account for the appearance of the oscillations. Indeed, after disassembly the backing was only partially bonded to the PDMS, falling off altogether after being lightly touched. Even after incurring optical damage, the peak-to-peak pressure of the signal remains 96% of that before damage at 159.29 μJ, though the quality of the signal is more severely impacted. As this transmitter already had a spike due to a somewhat thick glue layer, it may be that it was more damage-prone than a transmitter with a thinner layer where the backing is more securely bonded. On this basis illuminating the transmitters with fluence values below 15 mJ cm^−2^ should allow for damage-free operation.

**Fig. 8:**
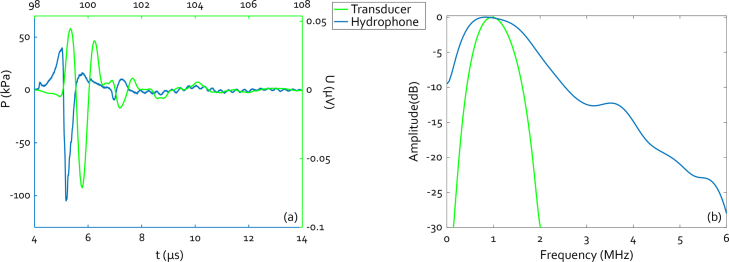
(a) Representative on-axis time signals for 950 μJ pulse energy. The bottom and left axes, in blue, correspond to the hydrophone-measured signal while the top and right axes correspond to that measured by the transducer.(b) Frequency spectra corresponding to both time signals.

For further measurements, using an undamaged transmitter, a liberal safety margin to prevent damage was maintained. The transmitter was illuminated with pulse energies of 950 μJ, giving an approximate fluence of 9.3 mJ cm^−2^. Fig. 8 shows the on-axis waveforms and spectra as they typically appear, measured at a depth of 7.42 mm by fibre-optic hydrophone and 147 mm by the custom US transducer mentioned in Section 2.1. The hydrophone measured LIUS signal shows no spike in the falling edge, indicating better adhesion of the backing. The secondary pulse at 7.5μs, from the internal reflection, has a peak-to-peak amplitude of 13.5% that of the main peak in both the hydrophone and the transducer signals. The peak-to-peak pressure is 144.73 kPa, from a maximum of 39.83 kPa to a minimum of −104.9 kPa. The spectrum of the hydrophone measurement has a −6 dB bandwidth of 1.88 MHz from 0.17 to 2.05 MHz, the centre frequency is 0.94 MHz, the maximum is located at 0.85 MHz. As measured with the custom transducer the spectrum peaks at 0.97 MHz, with a 98.9% bandwidth from 0.52 to 1.48 MHz. The peaks in both spectra overlap well, meaning that the signals can also be detected with adequate efficiency by the more narrowband detector. The slight deviation of the centre frequency from the model may be attributable to the absence of acoustic attenuation in the design model, something which can be improved in future iterations.

**Fig. 9:**
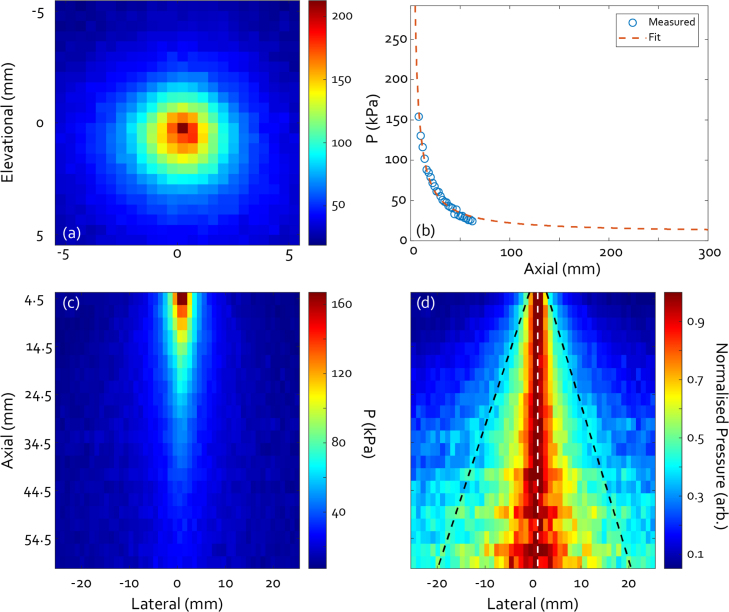
(a) Face-on pressure map at 8 mm distance from the front face of the transmitter, showing the symmetry of the field. (b) On-axis axial scan of peak-to-peak pressure (blue circles) and fitted function ∝$r^{-1}$. (c) Pressure map in the horizontal plane containing the acoustic axis and (d) row-by-row normalised version of the same, including dashed lines indicating the acoustic axis and e^−1^ cut-off, encompassing the far-field opening angle of 45.8${}^{\circ }$.

Fig. 9(a) shows a hydrophone scan of the emitted pressure field in the plane perpendicular to the acoustic axis, here referred to as the XY-plane. Fig. 9(b) shows the on-axis pressure decay with axial depth together with a fit ∝$r^{-1}$. The fit allows us to estimate the pressure at greater depths than 80 mm, beyond which point the hydrophone sensitivity was not sufficient to practically detect the signal. From the fit, the estimated pressure at 300 mm depth would be around 10 kPa. Even after transmission through a full 30 cm of tissue (α=2 db cm^−1^ [31]), this would still leave a pressure of 10 Pa, well above the minimum detectable pressure of our custom transducer. Fig. 9(c) and (d) show the pressure map in the horizontal plane containing the acoustic axis, here referred to as the XZ-plane, in kPa and normalised row by row respectively. The pressure maps, together, show the axisymmetric nature of the acoustic field as well as its behaviour with increasing depth. The black dashed lines in (d) indicate a fitted line to the *e*^−1^ points at each depth, from which the opening angle of 45.8${}^{\circ }$ was derived. The white dotted line in (d) indicates the acoustic axis. These hydrophone scans took about 1.5 h to complete, illuminating with 970 μJ pulse energy at a 100 Hz repetition rate. This entailed an estimated total of $5.4\cdot 10^{5}$ laser pulses fired at the transmitter, after which there was no noticeable degradation of the signal quality. This gives some indication of the potential for long-term stable operation of the LIUS transmitters.

### 3.4 General discussion

The LIUS transmitters presented in this work are still very much prototypes. Were they to be made for wider distribution and application some further improvements would be advised. The glass backing is quite fragile in the current design, meaning even fairly light mechanical pressure applied to the front face of the transmitter can lead it to break or detach. This immediately deteriorates the signal quality, if not necessarily the pressure, in unpredictable ways. One way to make it more robust would be to have the backing not simply attached to the PDMS, but integrated with the casing. This could be done by using a wider glass disc or machining the PMMA casing to incorporate a thin layer of material on top of the air pocket. To lower the chances of the backing detaching a different bonding mechanism may also be considered. In microfluidics highly robust bonding of PDMS is regularly performed to both glass, through oxygen plasma treatment [32], and PMMA, by treatment with 3-aminopropyltriethoxysilane [33]. Such a bonding method has the added bonus that it does not introduce an extra layer of a different material, however thin it may be, to the transmitter as the use of cyanoacrylate does. The main motivation for not doing this in this work is that various materials and shapes for the backing could be more rapidly tested by having a quick, simple bonding method that does not require any extra infrastructure to apply. For future iterations of these LIUS transmitters it is highly recommended to apply the mentioned surface modification bonding methods for a more robust result.

While PMMA is a low-cost material that is also easy to machine, a further simplification of the fabrication process might be achieved by 3D-printing the casings instead. As long as the precision is high enough to allow for the placement of the optical fibre with variations of no more than ≈200 μm and the PDMS can be bonded to the material, this should not be problematic. Screw threads can also be printed [34], meaning it should be possible to print the entire casing in just 2 pieces, the main casing and the fibre clamp, greatly simplifying the whole process. While this article presents a general approach to designing and fabricating a range of LIUS transmitters with different responses, we only show a single type. In future work it would be good to test the approach for various different frequency ranges to get a more complete outlook on the reliability of the process.

As described above, the current method of fabrication includes a considerable potential for variability in transmitter properties, due to the hand-made nature of the transmitters. In applications using multiple transmitters in concert, it is therefore important to characterise each transmitter well, and to be sure that their properties do not change during an measurement. Even if there is an appreciable variation in transmitter pressure output, or even centre frequency, knowing exactly what these variations are will let them be taken into account. Ideally, in future improvements to the fabrication process any variability would be minimised by more standardised and automated fabrication steps, as well as by ensuring robust bonding methods that do not vary with time and are not susceptible to optical damage in the course of normal operation.

The calibrated fibre-optic hydrophone used for the characterisation measurements was well-suited to determining the field geometry, the active sensor having a diameter of 10μm and the associated wide acceptance angle. This does, however, limit the sensitivity of the sensor and thus its ability to detect lower-pressure signals at greater depths. The addition of a larger, more sensitive, calibrated sensor for long-distance measurements would have allowed a more precise assessment of the transmitter’s performance at such depths needed for large-volume USCT measurements. As it stands, the fit to the data in Fig. 9(b) seems to give an adequate estimate of performance, though future measurements would bolster this conclusion.

For integration into a hemispherical large-volume PAT/USCT system the positioning of the transmitters needs to be considered carefully. Depending on the requirements of the PAT illumination and the density of detecting elements, a minimum number of LIUS elements for sufficient-quality images should be arrived at. Considering the desire to insonify as much of the imaging target as possible with each LIUS shot, it is likely that a narrow ring nearer the rim of the bowl would be optimal. In this manner a large range of angular positions could be covered by perhaps a few tens of transmitters. With such a number of transmitters in mind, the illumination method should also be addressed. One method to efficiently illuminate such an array of LIUS transmitters sequentially could be through the use of a fibre-optic multiplexer based on a matrix of fibre inputs, selectively illuminated by a laser via galvanometer mirrors and a scanning lens, as we have presented in previous work [8].

A final consideration for the integration of the LIUS transmitter into a multimodal system with a PAT capability is the generation of parasitic signals. When illuminating the imaging volume for PAT measurements, light hitting the front face of the LIUS transmitters is absorbed and can generate unwanted signals orders of magnitude stronger than the actual PA signals. This is highly undesirable and has to be addressed by preventing optical absorption in the front face of the LIUS transmitters. One of the easiest ways to do this, with minimal impact on the LIUS signal quality, would be to place gold foil stickers on the front faces of the transmitters, which would reflect most of the light and drastically, if not completely, reduce parasitic signals. A similar approach has already been shown to work in preventing parasitic signals from optically absorbing ultrasound detectors in a photoacoustic mammoscope [35].

For future work, several options present themselves to further improve control over the output of the LIUS transmitters. For one, the addition of a broadband acoustic matching layer [36], [37] to the front face of the transmitter would allow increased energy transmission and diminish or remove the internal reflection peak. The same could be achieved with a simple quarter-wave layer of an impedance-matching material like PDMS with embedded TiO${}_{2}$ [29] if the bandwidth of the generated signal could somehow be further reduced, which may be desirable depending on the detector being used. Further control over the frequency spectrum of the transducer could be achieved both by altering the laser pulse duration and by changing the physical parameters of the absorbing material [38]. In the former increasing the optical pulse duration up to values on par with the stress confinement time will broaden the generated acoustic pulse and thus decrease the bandwidth and centre frequency. One can imagine matching optical absorption properties to laser pulse duration and temporal profile to attain a range of desired spectral responses. In the latter the deposition of multiple low-absorbing layers of any combination of thicknesses and optical properties may also serve to shape the pulse and thereby also shape the spectrum.

## 4 Conclusion

We have presented an approach to designing laser-induced ultrasound transmitters for large-volume ultrasound computed tomography. The transmitters produced according to our method behave in a satisfactory and reproducible manner, though some room for improvement in the process and result still remains. The LIUS transmitters produced here have a centre frequency of 0.94 MHz with a bandwidth from 0.17 to 2.05 MHz, producing pressures between 180.17 kPa and 24.35 kPa for a range of depths between 7.42 and 62.25 mm. The ultrasound field has a cylindrical symmetry about the acoustic axis and an opening angle of 45.8${}^{\circ }$. These design and fabrication methods can be used to create a wide range of LIUS transmitters for any number of applications.

## Declaration of Competing Interest

The authors declare that they have no known competing financial interests or personal relationships that could have appeared to influence the work reported in this paper.

## References

[1] Bychkov A., Simonova V., Zarubin V., Cherepetskaya E., Karabutov A. (2018). The progress in photoacoustic and laser ultrasonic tomographic imaging for biomedicine and industry: a review. Appl. Sci..

[2] Chen S.-L. (2017). Review of laser-generated ultrasound transmitters and their applications to all-optical ultrasound transducers and imaging. Appl. Sci..

[3] Karabutov A.A., Savateeva E.V., Podymova N.B., Oraevsky A.A. (2000). Backward mode detection of laser-induced wide-band ultrasonic transients with optoacoustic transducer. J. Appl. Phys..

[4] Pelivanov I., Ambroziński Ł., Khomenko A., Koricho E.G., Cloud G.L., Haq M., O’Donnell M. (2016). High resolution imaging of impacted CFRP composites with a fiber-optic laser-ultrasound scanner. Photoacoustics.

[5] Ambrozinski L., Mrowka J., O’Donnell M., Pelivanov I. (2019). Detection and imaging of local ply angle in carbon fiber reinforced plastics using laser ultrasound and tilt filter processing. Composites A.

[6] Alles E.J., Noimark S., Zhang E., Beard P.C., Desjardins A.E. (2016). Pencil beam all-optical ultrasound imaging. Biomed. Opt. Express.

[7] Colchester R.J., Mosse C.A., Bhachu D.S., Bear J.C., Carmalt C.J., Parkin I.P., Treeby B.E., Papakonstantinou I., Desjardins A.E. (2014). Laser-generated ultrasound with optical fibres using functionalised carbon nanotube composite coatings. Appl. Phys. Lett..

[8] Thompson D., Kruit H., Gasteau D., Manohar S. (2020). Laser-induced synthetic aperture ultrasound imaging. J. Appl. Phys..

[9] Alles E.J., Noimark S., Maneas E., Zhang E.Z., Parkin I.P., Beard P.C., Desjardins A.E. (2018). Video-rate all-optical ultrasound imaging. Biomed. Opt. Express.

[10] Thompson D., Gasteau D., Manohar S. (2020). Spatially compounded plane wave imaging using a laser-induced ultrasound source. Photoacoustics.

[11] Wurzinger G., Nuster R., Paltauf G. (2016). Combined photoacoustic, pulse-echo laser ultrasound, and speed-of-sound imaging using integrating optical detection. J. Biomed. Opt..

[12] Du X., Li J., Niu G., Yuan J.-H., Xue K.-H., Xia M., Pan W., Yang X., Zhu B., Tang J. (2021). Lead halide perovskite for efficient optoacoustic conversion and application toward high-resolution ultrasound imaging. Nature Commun..

[13] Colchester R.J., Little C., Dwyer G., Noimark S., Alles E.J., Zhang E.Z., Loder C.D., Parkin I.P., Papakonstantinou I., Beard P.C. (2019). All-optical rotational ultrasound imaging. Sci. Rep..

[14] Chen Y., Li Q., Zhu H., Wang Y., Zhang X., Yu H. (2021). Air-backed photoacoustic transmitter for significantly improving negative acoustic pressure output. Opt. Lett..

[15] Li Q., Li J., Zhu H., Chen Y., Zhu B., Yu H. (2021). Dynamic acoustic focusing in photoacoustic transmitter. Photoacoustics.

[16] Heo J., Biswas D., Park K.K., Son D., Park H.J., Baac H.W. (2021). Laser-generated focused ultrasound transducer using a perforated photoacoustic lens for tissue characterization. Biomed. Opt. Express.

[17] Hsieh B.-Y., Chen S.-L., Ling T., Guo L.J., Li P.-C. (2012). All-optical scanhead for ultrasound and photoacoustic dual-modality imaging. Opt. Express.

[18] Ermilov S., Su R., Conjusteau A., Anis F., Nadvoretskiy V., Anastasio M., Oraevsky A. (2016). Three-dimensional optoacoustic and laser-induced ultrasound tomography system for preclinical research in mice: design and phantom validation. Ultrason. Imaging.

[19] Joo M.G., Lee K.-T., Sang P., Heo J., Park H.J., Baac H.W. (2019). Laser-generated focused ultrasound transmitters with frequency-tuned outputs over sub-10-MHz range. Appl. Phys. Lett..

[20] Gasteau D., Thompson D., Nagel J., Van Hespen J., Manohar S. (2020). Photons Plus Ultrasound: Imaging and Sensing 2020, Vol. 11240.

[21] Zhou Y., Yao J., Wang L.V. (2016). Tutorial on photoacoustic tomography. J. Biomed. Opt..

[22] Karabutov A., Podymova N., Letokhov V. (1996). Time-resolved laser optoacoustic tomography of inhomogeneous media. Appl. Phys. B.

[23] Abrarov S.M., Quine B.M. (2018). A rational approximation of the Dawson’s integral for efficient computation of the complex error function. Appl. Math. Comput..

[24] Rwei S.-P., Manas-Zloczower I., Feke D. (1990). Observation of carbon black agglomerate dispersion in simple shear flows. Polym. Eng. Sci..

[25] Albers P., Seibold K., Prescher G., Freund B., Parker S., Tomkinson J., Ross D., Fillaux F. (1999). Neutron spectroscopic investigations on different grades of modified furnace blacks and gas blacks1Parts of this paper were presented at the 23rd Biennial Conference on Carbon, July 13–18 1997 at the Pennsylvania State University, State College, USA, Extended Abstracts pp. 590–591.1. Carbon.

[26] Cox A., DeWeerd A.J., Linden J. (2002). An experiment to measure Mie and Rayleigh total scattering cross sections. Amer. J. Phys..

[27] Janzen J. (1979). The refractive index of colloidal carbon. J. Colloid Interface Sci..

[28] Cafarelli A., Verbeni A., Poliziani A., Dario P., Menciassi A., Ricotti L. (2017). Tuning acoustic and mechanical properties of materials for ultrasound phantoms and smart substrates for cell cultures. Acta Biomater..

[29] Guillermic R.-M., Lanoy M., Strybulevych A., Page J.H. (2019). A PDMS-based broadband acoustic impedance matched material for underwater applications. Ultrasonics.

[30] Xu G., Ni Z., Chen X., Tu J., Guo X., Bruus H., Zhang D. (2020). Acoustic characterization of polydimethylsiloxane for microscale acoustofluidics. Phys. Rev. A.

[31] Bamber J.C., Hill C. (1979). Ultrasonic attenuation and propagation speed in mammalian tissues as a function of temperature. Ultrasound Med. Biol..

[32] Jo B.-H., Van Lerberghe L.M., Motsegood K.M., Beebe D.J. (2000). Three-dimensional micro-channel fabrication in polydimethylsiloxane (PDMS) elastomer. J. Microelectromech. Syst..

[33] Vlachopoulou M., Tserepi A., Pavli P., Argitis P., Sanopoulou M., Misiakos K. (2008). A low temperature surface modification assisted method for bonding plastic substrates. J. Micromech. Microeng..

[34] MacLeod A., Patterson M., MacTear K., Gill H.S. (2020). 3D printed locking osteosynthesis screw threads have comparable strength to machined or hand-tapped screw threads. J. Orthopaedic Res.®.

[35] Schoustra S.M., Piras D., Huijink R., Op’t Root T.J., Alink L., Kobold W.M.F., Steenbergen W., Manohar S. (2019). Twente Photoacoustic Mammoscope 2: system overview and three-dimensional vascular network images in healthy breasts. J. Biomed. Opt..

[36] Li Z., Yang D.-Q., Liu S.-L., Yu S.-Y., Lu M.-H., Zhu J., Zhang S.-T., Zhu M.-W., Guo X.-S., Wu H.-D. (2017). Broadband gradient impedance matching using an acoustic metamaterial for ultrasonic transducers. Sci. Rep..

[37] Martin B. (1992). A note on multilayer acoustic antireflection coatings. Res. Nondestruct. Eval..

[38] Rajagopal S., Cox B.T. (2021). Modelling laser ultrasound waveforms: The effect of varying pulse duration and material properties. J. Acoust. Soc. Am..

